# Hepatitis B viremia manifesting as polyarteritis nodosa and secondary membranous nephropathy

**Published:** 2016-01-14

**Authors:** Manish Rameshlal Balwani, Vivek B Kute, Pankaj R Shah, Maulin Shah, Saiprasad G Shinde, Jay Shah, Hargovind L Trivedi

**Affiliations:** ^1^Department of Nephrology and Clinical Transplantation, Institute of Kidney Diseases and Research Center, Dr. HL Trivedi Institute of Transplantation Sciences (IKDRC-ITS), Ahmedabad, India

**Keywords:** Membranous nephropathy, Hepatitis B, Polyarteritis nodosa, Plasmapheresis

## Abstract

Renal involvement in hepatitis B-polyarteritis nodosa (HBV-PAN) usually occurs in the form of hypertension, microscopic hematuria, proteinuria but nephrotic range proteinuria or renal failure is very uncommon. A 60-year-old man had abdominal pain for 15 days which was followed by bilateral pedal edema in a day and in next three days he had sudden onset bilateral foot drop with numbness. He had weight loss of 10 kg in last 20 days. Pedal edema was pitting, bilateral. Macular skin rashes around both ankles were present. Serum creatinine was 2.4 mg/dl and blood urea nitrogen was 102 mg/dl.24 hour proteinuria was 3.4 g/day. Serum HBsAg, HBeAg and anti-HBc IgM were positive. Serum HBV-DNA level (PCR) was 582917 copies/ml. The nerve conduction study showed axonal and demyelinating polyneuropathy in bilateral lower limbs. A kidney biopsy revealed membranous nephropathy (MN). Doppler for renal vessels was normal. Prednisolone (60 mg/day), plasmapheresis (5 courses) and entecavir (0.5 mg/ every second day) were started. At 2 months follow up, patient improved in the form of grade 3/5 power in both lower limbs with sensory improvement. Twenty-four hours proteinuria decreased to 800 mg/day and serum creatinine reached to 1.5 mg/dl. Polyarteritis nodosa was due to HBV infection, but the etiology of MN was uncertain, as it has rarely been described in PAN. Proteinuria responded to nucleoside analogue therapy. So patient was considered to have an association of classic PAN and MN, both related to HBV. Patient responded to combined treatment of steroid, plasmapheresis and nucleoside analogue.

**Implication for health policy/practice/research/medical education:**

We report a severe case of polyarteritis nodosa (PAN) which developed after acute hepatitis B virus (HBV) with neurologic and renal involvement. The interesting aspect was the presence of concomitant membranous nephropathy (MN) and PAN secondary to HBV viremia. Both of conditions improved after starting specific antiviral drug entecavir. In our case, plasmapheresis and short course of steroid treatment were found to be effective in treatment of PAN. Thus we conclude that renal biopsy should be performed in a patient of acute HBV PAN with normal renal vessel imaging who presents with nephrotic range proteinuria to exclude secondary glomerulopathies.

## Introduction

Polyarteritis nodosa (PAN) usually is seen weeks to months after an acute hepatitis B virus (HBV) infection (1). Renal involvement in hepatitis B-polyarteritis nodosa (HBV-PAN) usually occurs in the form of hypertension, microscopic hematuria, and proteinuria, however nephrotic range proteinuria or renal failure is very uncommon (2,3). The renal pathology in PAN is usually a medium-sized arteritis with only ischemic changes in glomeruli. Here we report the presence of concomitant membranous nephropathy and PAN secondary to HBV viremia.

## Case presentation

**Figure 1:**
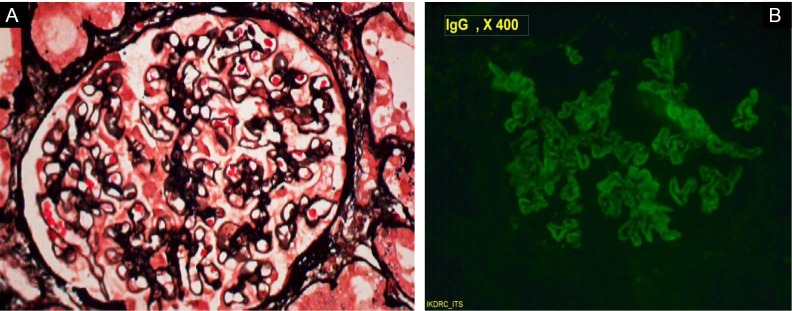
A) Silver stain showing thickened glomerular basement membrane suggestive of MN. B) Immunofluorescence stain showing coarse granular IgG deposition along the basement membrane(X 400 in both A & B).

A 60-year-old man was referred to our hospital for raised serum creatinine. The patient had abdominal pain for 15 days, which was mild, vague and non-radiating. It was followed by bilateral pedal edema in a day and in next three days followed by sudden onset bilateral foot drop with numbness. Patient gave history of nausea, vomiting, skin rash, anorexia and malaise since 20 days. He had weight loss of 10 kg in last 20 days. The past medical history was unremarkable. Alcohol consumption and drug abuse were absent. On physical examination, temperature was 38.4°C, blood pressure was 160/100 mm Hg, pulse rate 84/min and respiratory rate 20/min. Pedal edema was pitting, bilateral. Macular skin rashes around both ankles were present. Testicular tenderness was present. There was sensory and motor involvement in bilateral tibia and peroneal nerve distribution. Funduscopic examination showed grade 3 hypertensive retinopathy. Laboratory examination revealed a hemoglobin of 12.1 g/dl, white blood cell count of 24000/µl, platelet count of 485000/µl. Serum creatinine was 2.4 mg/dl, blood urea nitrogen 102 mg/dl, potassium 5.0 mEq/l, albumin 3.1 g/dl, aspartate aminotransferase 27 U/l, alanine aminotransferase 20 U/l, alkaline phosphatase 131 U/l and total cholesterol 198 mg/dl. Prothrombin and partial thromboplastin times were normal. Urinalysis revealed proteinuria, with two to three oval fat bodies per high-power field. Protein excretion was 3.4 g/day. Bence jones proteinuria, urine immunoelectrophoresis showed non selective proteinuria. On admission, HBsAg, HBeAg and anti-HBc IgM were positive. Anti-HBs, anti-HBe, anti-HCV and anti-HIV were negative. Serum HBV-DNA level (PCR) was 582917 copies/ml. ANA, anti-dsDNA, RF, p-ANCA, c-ANCA, and cryoglobulins were negative. Serum C3 and C4 complement levels were normal. Chest x-ray and electrocardiography were normal. The nerve conduction study was compatible with axonal and demyelinating polyneuropathy in bilateral lower limbs. A kidney biopsy was performed due to nephrotic range proteinuria and revealed membranous nephropathy (MN). All glomeruli were mildly enlarged. Capillary lumina were open and lined by thickened membranes and there was spikes on the external side of the glomerular basement membrane (GBM) (Jones stain). Mild uniform matrix accentuation with normal cell distribution was noted. Tubules were moderately degenerated. Interstitial area was moderately prominent for edema with leucocyte infiltration. Immunofluorescence study, showed significant granular IgG deposition along the membranes of capillary in a diffuse pattern (Figure 1). There was no immunofluorescent staining for fibrinogen, IgA, or IgM. Congo-red staining for amyloid was negative. A liver biopsy was not performed. Doppler study for renal vessels was normal and there were no microaneurysms characteristic of PAN. Prednisolone (60 mg/day), plasmapheresis (5courses) and entecavir (0.5 mg/ every second day) were started. At 2 months follow up, patient improved in the form of grade 3/5 power in both lower limbs with sensory improvement. Twenty-four hours proteinuria decreased to 800 mg/day and serum creatinine was 1.5 mg/dl with no pedal edema.

## Discussion

Acute HBV-PAN usually is seen weeks to months after an acute HBV infection (1). Renal involvement in HBV-PAN usually occurs in the form of hypertension, microscopic hematuria, proteinuria but nephrotic range proteinuria or renal failure is very uncommon (2,3). We present a patient who developed PAN in the early course of acute HBV, while, this is probably the first reported case because of the presence of simultaneous nephrotic syndrome due to secondary MN. A putative diagnosis of PAN was made based on his clinical symptoms. He met eight of the 10 criteria of the American College of Rheumatology, namely weight loss of 10 kg, myalgia, weakness, skin rashes, neuropathy, diastolic blood pressure >90 mm Hg, altered renal function test, HBV positivity and testicular tenderness (4). Guillevin et al (5) suggested the five-factors score (FFS) which is a good predictor of death. They include the factors such as increased serum creatinine, proteinuria, cardiomyopathy, and gastrointestinal involvement and central nervous system signs. Mortality at 5 years was 46% when FFS was equal to or more than 3, and our patient fulfilled 3 of these factors. Frequency of HBV-PAN has decreased over the past 10 years (6). Most cases are diagnosed after anti-HBc IgM becomes negative (4,7). In our case, PAN developed when the patients anti Hbs IgM and HBV DNA by PCR was positive. Renal involvement is seen in 70%–100% of cases. The renal pathology in PAN is usually a medium-sized arteritis with only ischemic changes in glomeruli (8). Our patient had nephrotic-range proteinuria and renal biopsy disclosed membranous glomerulopathy. Presence of nephrotic syndrome in our case was attributed to increased glomerular permeability to protein probably due to HBV-associated MN. The mechanism of vasculitis in HBV-PAN is thought to be immune complex deposition. In PAN, high HBV viral replication might lead to continuous stimulation and the production of circulating immune complexes, which deposit on the vascular walls with complement fixation which will result in damage to the blood vessels, causing vasculitis (6). In chronic HBV infection corticosteroids and immunosuppressants was shown to enhance viral replication. Guillevin et al studied the outcome of patients treated with prednisone, vidarabine and plasma exchange for a short period which he found to be good (6,9). Plasmapheresis rapidly and efficiently removes antigen–antibody complexes, pathogenic antibodies and inflammatory mediators. Thus, it is particularly useful in several immune complex disorders, such as HBV-PAN (10). In our case we used steroids, plasmapheresis and entecavir. Five sessions of plasmapheresis were done, steroid in form of prednisolone started at 1 mg/kg/day was gradually tapered in a month. Entecavir was given at a dose of 0.5 mg/kg every other day. At follow up of two months, patient improved in the form of grade 3/5 power in both lower limbs with sensory improvement. Twenty-four hours proteinuria decreased to 800 mg/day and serum creatinine was 1.5 mg/dl. HBV-DNA levels were 11279 copies/ml by PCR method after 2 months of entecavir treatment.

## Conclusion

We report a severe case of PAN which developed after acute HBV with neurologic and renal involvement. The interesting aspect was the presence of concomitant MN and PAN secondary to HBV viremia. Both of conditions improved after starting specific antiviral drug entecavir. In our case, plasmapheresis and short course of steroid treatment were found to be effective in treatment of PAN. Thus we conclude that renal biopsy should be performed in a patient of acute HBV-PAN with normal renal vessel imaging who presents with nephrotic range proteinuria to exclude secondary glomerulopathies.

## Authors’ contribution

All authors wrote the paper equally.

## Conflicts of interest

The authors declared no competing interests.

## Ethical considerations

Ethical issues (including plagiarism, data fabrication, double publication) have been completely observed by the authors.

## Funding/Support

None.
